# Direct, Rapid Detection of Pathogens from Urine Samples

**DOI:** 10.3390/ma15217640

**Published:** 2022-10-31

**Authors:** Sorin David, Raluca-Elena Munteanu, Ana-Maria Tițoiu, Ionela-Cristina Petcu, Ioana-Cristina Cernat, Corina Leancu, Mihaela Gheorghiu, Eugen Gheorghiu

**Affiliations:** 1 International Centre of Biodynamics, Intrarea Portocalelor 1B, 060101 Bucharest, Romania; 2 Faculty of Biology, University of Bucharest, Splaiul Independenței 91-95, 050095 Bucharest, Romania; 3 Laboratoarele SynLab, Bld. Tudor Vladimirescu nr.29, 050881 Bucharest, Romania

**Keywords:** UTI, immunomagnetic separation, aggregates of magnetically tagged pathogens, rapid identification

## Abstract

The problem of rapidly detecting pathogens directly from clinical samples poses significant analytical challenges. Addressing this issue in relation to urinary tract infections, we propose an effective protocol and related immunomagnetic test kits enabling versatile screening for the presence of pathogenic bacteria in unprocessed urine samples. To achieve this, the components of a typical immunomagnetic separation protocol were optimized towards the sensitive assessment of the aggregates formed out of immunomagnetically tagged target pathogens collected from clinical samples. Specifically, a dedicated immunomagnetic material was developed via the functionalization of standardized, micron-sized magnetic beads with generic antibodies against gram-specific bacterial constituents with mannan binding lectin. As such, we demonstrate efficient procedures for achieving the enhanced, specific, and pathogen-mediated cluster formation of these tailored affinity-coated magnetic beads in complex samples. We further show how cluster analysis, in conjunction with the use of nonspecific, inexpensive fluorescent dye, allows for a straightforward optical assessment of the bacterial load directly from urine samples. The optimized sensing protocol and related kits provide, in less than 60 min, qualitative (positive/negative) information on the bacterial load with 85% specificity and 96% sensitivity, which is appropriate to empower clinical microscopy with a new analytic dimension. The procedure is prone to automation, can be conveniently used in clinical microbiology laboratories and, since it preserves the viability of the captured bacteria, can be interfaced with downstream analyses and antimicrobial susceptibility testing. Moreover, the study emphasizes a suite of practical validation assays that are useful for bringing the tool-box of immunomagnetic materials outside the academic laboratory and into real-life applications.

## 1. Introduction

The problem of the direct, rapid detection of pathogens from clinical samples is a matter of intense scrutiny. Urinary tract infection (UTI), the condition caused by the presence of pathogenic bacteria in the urinary tract (urethritis, cystitis, pyelonephritis), remains one of the most common infections, with approximately 150 million people affected each year [1]. UTIs can cause serious sequelae, including frequent recurrences, renal damage in young children, pre-term birth in pregnant women, and complications caused by the frequent use of antimicrobial drugs, including high-level antibiotic resistance. Moreover, research has shown that the source of infection in 20–30% of all patients with sepsis is localized in the urogenital tract and that urosepsis may cause mortality rates of 25% to 60% in certain patient groups [2]. Whereas gram-negative bacterial infections appear to dominate the UTI spectrum, the bacterial species that can cause UTIs are numerous and belong to both gram-negative and gram-positive genera such as *Escherichia coli*, *Klebsiella* spp., *Proteus* spp., *Pseudomonas aeruginosa* (gram-negative), *Streptococcus agalactiae*, and *Staphylococcus saprophyticus* (gram-positive).

To date, the gold standard for diagnosing UTIs in the presence of clinical symptoms is the detection and quantification (determination of bacteriuria) of the pathogen via the urine culture using the clean-catch midstream urine samples [3]. Identifying and quantifying the pathogens present in a given sample by culture is time- and resource-consuming. Faster (<24 h), orientating indirect methods (e.g., urine microscopy or dip sticks) are often used in practice to detect the presence of bacteria or inflammation. However, the bacterial count that may be assessed by urine microscopy or the optical density (OD) changes of special media measured by spectrophotometry are often outside the relevant limits or suffer from low specificity and sensitivity. The minimum level of bacteriuria demonstrating an infection of the urinary tract depends on the type of bacteria detected, varying from 10^3^ colony forming units (CFU)/mL to 10^5^ CFU/mL urine (the typical threshold [4]), and its rapid assessment is analytically challenging. These aspects often lead to the empirical selection of antibiotics [4]. Lateral flow immunoassays (e.g., RapidBac test [5], which can detect mostly gram-negative bacteria) are typically used as rapid assays in clinical settings; however, their qualitative nature, limited sensitivity, and lack of bacterial recovery options for further analysis are important drawbacks. Moreover, the clinical guidelines for UTIs indicate that antibiotics should be selected only when the antimicrobial susceptibility testing (AST) has been performed and the pathogen has been determined [6].

Thus, the problem of identifying bacteria early and in the lowest relevant concentrations while preserving bacterial integrity for subsequent AST is a pressing challenge for the appropriate management of UTIs in order to have a proper medical phenotypic diagnosis of complicated cases to reduce the empirical antimicrobial regimen of choice and effectively prevent the emergence of multi-drug-resistant uropathogens.

Addressing this problem, we propose the deployment of an optimized immunomagnetic separation procedure coupled with a nonspecific fluorescent dye for direct use in urine samples. Such an approach is able to provide the necessary boost for urine microscopy or optical density OD assays in terms of specificity and sensitivity, and it could form the basis for the development of fast pathogen detection and AST assays. To this end, we report on the development, testing, and validation of real urine samples of an immunomagnetic procedure comprising optimally functionalized magnetic particles for effective pathogen capturing and the formation of aggregates of magnetically tagged cells, allowing for a straightforward assessment of the bacterial load (positive/negative) via optical analysis.

Analytical technologies based on magnetic beads (MBs) coated with specific molecules offer a rapid, effective, and specific way to capture, separate, collect, and concentrate the target analytes from the sample matrix prior to detection. The method was proven to be effective in separating various types of samples, from the preconcentration of ions to the rapid, selective capture of organic compounds, biomolecules, and cells [7,8]. As a result, immunomagnetic separation (IMS) techniques are widely used in many sensing-related assays. For example, a method based on fluorescence microscopy that is capable of detecting *E. coli* bacteria in a buffer with a system based on magnetic glycol-particles is described by El-Boubbou and coworkers [9]. Another approach where magnetic particles enabled an advantage over molecular methods is described by Dogan et al. [10]. In this study, a combination of immunomagnetic separation and fluorescence techniques (based on Quantum Dots, QD) was reported for the enumeration of *E. coli* from spiked urine samples, with a total analysis time less than 120 min. Of note, immunomagnetic separation and absorbance measurements for bacterial target analyte detection were, to our knowledge, not decoupled from the enzymatic conversion of chromogenic substrates to optically measured products [11,12,13,14,15,16], while an increased sensitivity and amenability to point of care (POC) formats were demonstrated only when coupled to fluorescence assays and specially designed immunomagnetic particles [16,17]. Our group’s previous results [18] showed, at the proof-of-concept level in synthetic samples, avenues for the improved, selective capture of the target bacteria from complex samples in addition to the formation of sizeable cells–magnetic beads clusters with morphologies and structures dependent on the target cells number. Optimizing materials for the immunomagnetic isolation and microscopic analysis of immunomagnetic clusters is an actual trend [19,20,21]; yet, their implementation in minimally processed samples is as of yet an unmet need.

Particle-based configurations and immune-recognition not only allow for rapid, selective cell capture from clinical samples but are also effective for subsequent analysis via microfluidic systems’ optical and electrical assays [22] or even matrix-assisted laser desorption/ionization time-of-flight mass spectrometry, and they thus emerge as viable alternatives to molecular approaches [23,24] and culture-based methods for quantifying the amount of bacteria and performing physiomics analysis. Although IMS provides simple, rapid, sensitive, and low-cost methods for the isolation of target organisms, the sample matrix always interferes with the capture of bacteria by immunomagnetic particles, resulting in altered capture efficiencies (CE), reduced sensitivity, and a lack of validation. In most reported assays, the actual identification and quantitation of the captured cells is subsequently performed by classical plate counts or microbiological assays. Thus, challenges to overcome this concern the optimization of the capture efficiency of immunomagnetic protocols, the evaluation of cell vitality (ability to proliferate) once immunomagnetically captured, and method validation.

In this paper, we report on end-user (i.e., clinical microbiology laboratories)-oriented ways to solve these challenges and provide a validated procedure for the rapid, selective evaluation of the specific bacterial load of clinical samples related to UTI. The focus is placed on the optimization of immunomagnetic materials, i.e., the smart functionalization of commercial magnetic beads to cover a wide spectrum of causative agents (with both gram-positive bacteria as well as yeasts that may be present in urine samples), and affordable and clinically available set-ups coupled with tests in parallel with an accredited clinical microbiology laboratory (SynLab, Romania) to validate the results. As such, the IMS-proposed protocol (the use of the immunomagnetic procedure in conjunction with optical evaluation) validated using actual clinical samples is more than an enrichment strategy and an orientating, qualitative, method for rapid detection; it could also enable progress towards a fast decision tool for antibiotic treatment selection in a timely manner and facilitate its smooth clinical integration and acceptance.

## 2. Materials and Methods

### 2.1. Chemicals

Magnetic beads (Dynabeads™ M-270 Carboxylic Acid, 30 mg/mL) purchased from Invitrogen Life Technologies (Waltham, MA, USA)were used for the development of immunomagnetic particles in combination with three different antibodies for immune recognition: Anti-*E. coli* O + *E. coli* K (ab31499) Rabbit polyclonal antibody against all O and K antigenic serotypes of *E.coli*, Mouse monoclonal antibody against gram-positive bacteria (ab267414/ab20344), which reacts with gram-positive bacteria Lipoteichoic acid (LTA), and Mouse monoclonal antibody against gram-negative Endotoxin (ab41201) as well as a Human Mannan Binding Lectin/MBL peptide (237–248) (Carboxyterminal end), which were all purchased from Abcam, Cambridge, UK.

Bacto™ Peptone (from BD Biosciences, San Jose, CA, USA), yeast extract, NaCl (Sigma-Aldrich), 2-[N-morpholino] ethanesulfonic acid (MES), 1-ethyl-3-(3-dimethyl-aminopropyl) carbodiimide (EDC), N-hydroxy-succinimide (NHS), Tween 20, Phosphate Buffer Saline (PBS) pH 7.4, NaH_2_PO_4_, Na_2_HPO_4_, and KCl were purchased from Sigma Aldrich. Acridine orange hydrochloride hydrate (AO) was purchased from Merk. Ultrapure water (18.2 MΩ·cm) was obtained from a Millipore Direct-Q system.

### 2.2. Reference Bacteria Culture

The bacterial reference strain used in the development of the assay was *Escherichia coli* ATCC 25922 (serotype O6, Biotype 1), which was provided by the Microbiology Laboratory, University of Bucharest. The cells were maintained on solid culture media and transferred to liquid media 24 h before the experiment. A minimal growth medium containing 10 g Bacto Peptone, 5 g yeast extract, 3.5 g Na_2_HPO_4_, 1.5 g KH_2_PO_4_, and 5 g NaCl in 1 L of ultrapure water was chosen for the reference *E. coli* cultivation. The pH of the cultivation medium was adjusted at 7.2 ± 0.2 before sterilization in an autoclave. After inoculation, the bacteria culture was grown for at least 16 h on a shaker at 37 °C.

The turbidity of the cell cultures was adjusted based on McFarland standards using a spectrophotometer (λ = 620 nm), and bacteria dilutions were prepared using sterile PBS.

Live *E. coli* cells at a concentration of 10^9^ CFU/mL are optionally mixed with AO (2 μg/mL concentration), and the cells are resuspended in PBS pH 7.4, with the supernatant removed by centrifugation. Successive 1:10 test dilutions in PBS were made from 10^8^ down 10^0^ CFU/mL.

### 2.3. Immunomagnetic Materials Preparation: Magnetic Beads Functionalization and Antibody Immobilization

For the synthesis of the immunomagnetic material, functionalization with different antibodies—Anti-*E. coli* O157:H7, Anti gram-positive, Anti gram-negative and a generic lectin Manan (Mannose Binding Lectin—MBL, hereafter)—was carried out, and specific blocking procedures were devised and implemented on commercial Magnetic beads, Dynabeads™ (30 mg/mL) with a 2.8 µm diameter, and carboxylic acid group M-270 Carboxylic Acid (CMB, hereafter)- or tosyl (TMB, hereafter)-activated surfaces. Adjusted manufacturer protocols (https://www.thermofisher.com/order/catalog/product/14305D, accessd on 10 July 2022) were followed to prepare stock solutions containing specific bioaffinity compounds. These include common steps and, for carboxylic acid group surfaces, an activation one. All MB-270 particles were vortexed for 30 s and submitted to ultrasonication for 30 min to ensure a very good dispersion of the suspension, followed optionally by an EDC/NHS surface activation step.

For the tosyl MB, the EDC/NHS activation step is not necessary; for M-270 Carboxylic Acid, this step consists of two rounds of MES 25 mM, pH = 5 washing followed by the 30 min-long activation step of carboxylic groups, using a mixture of 1:1 EDC/NHS (50 mg/mL in MES 25 mM) in a volume of 100 μL. The activated MBs are washed twice with MES and incubated for 30 min in a solution of protein G (1 mg/mL in MES). Upon two more rounds of washing with MES, the 30 min incubation in a solution of the desired bioaffine compound follows. Then, for the blocking step, we use 1 mg/mL of BSA solution in PBS: the magnetic particles are washed two times with the blocking solution and incubated for 60 min under agitation in 100 μL of the same solution. Finally, the functionalized MBs were washed and resuspended in PBS to a final 100 μL volume test stock. For every sample, a fixed 5 µL MB volume (corresponding to 10^7^ beads) was used. Immunomagnetic capture is acquired in 1.5 mL centrifuge vials and a bench top rotating mixer (Phoenix RS-RD-5 from Phoenix Instrument, Garbsen, Germany). To separate the magnetic particles from the solution, the vials are placed for 1 min next to a neodymium magnet. Further processing steps, characteristic of each employed analysis method, are described in the following sections.

The amount of bioaffinity compound (antibody) and the incubation time are set upon optimization for high-efficiency analyte capturing and for the formation of specific clusters.

### 2.4. Experiments for Assessing the Efficiency of the Immunomagnetic Capture

**Scheme 1:**
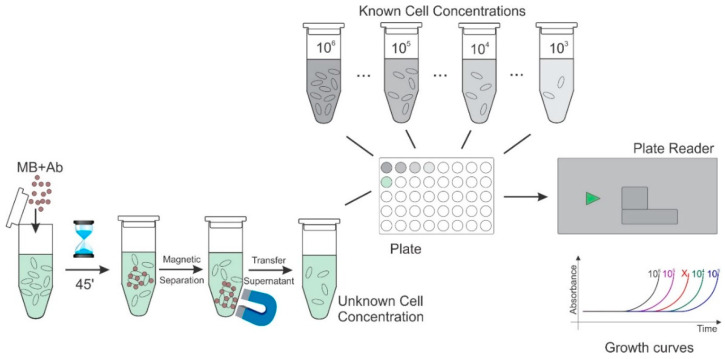
Steps of the protocol for the validation of MBs capture.

To determine the capture rate of the advanced immunomagnetic materials and the support method optimization, a high-throughput assay was implemented based on 96-well plate absorbance-derived growth curves of reference cultures. Measurements were performed on an Infinite F200 Pro plate reader (Tecan, Männedorf, Switzerland). The reference test samples undergo Scheme 1 steps prior to optical evaluation. According to these steps, the optimized immunomagnetic materials (e.g., MBs functionalized with affinity compounds) are incubated with reference samples (used as the control), undergo magnetic separation and the growth curves based on the absorbance changes of the cells in the supernatant, and are affinity-bound on the immunomagnetic materials.

To achieve IMS, the optimized immunomagnetic materials (e.g., MBs functionalized with specific *E. coli* antibody) are mixed with the reference samples, placed on a rotating mixer, and separated from the supernatant with a fixed magnet. Next, the growth curves of known (reference) *E. coli* concentrations, of the collected MB-*E. coli* clusters, and of the supernatant withdrawn before MB-*E. coli* clusters collection are evaluated based on absorbance data. The OD at 620 nm is assessed every 15 min for 16 h of cultivation in 1:1 diluted growth medium with PBS buffer, with the microplate continuously shaken between measurements, under a 37 °C incubation temperature.

Additionally, to evaluate the capture efficiency for *E. coli* cells in specific MB clusters, we used end-point assays and a fluorescence method based on AO staining evaluation with a Multimodal GloMax 20/20 Reader (Promega from Madison, WI, USA) luminometer equipped with a Blue fluorescence module (excitation, 460 nm; emission, 515–575 nm).

CE is typically defined as the percentage of the total number of cells retained on immunomagnetic particles versus a known concentration of the test culture, according to Equation (1) [25]:

(1) $$\mathrm{CE}\%=\left(1\;-\;\frac{C_{\sup}}{C_{\mathrm{cells}}}\right)\times \;100\;\;\mathrm{or}\;\mathrm{alternatively}\;\mathrm{CE}\%=\left(1\;-\;\frac{F_{\sup}}{F_{\mathrm{cells}}}\right)\times \;100$$

where C_sup_ is the concentration of the cells that were not immunomagnetically separated (remaining in the sample), and C_cells_ is the concentration of cells in the reference sample.

These amounts are typically evaluated by plate count, i.e., a procedure that is both time-consuming and imprecise. In contrast, a rapid fluorescence assay involving AO staining and the assessment of F_sup_ (the fluorescence of the cells that were not immunomagnetically separated) versus F_cells_ (the fluorescence of the cells undergoing immunomagnetic separation) is deployed to quantitatively evaluate these distinct parameters of immunomagnetic beads synthesis towards method optimization. To this end, specific calibration curves based on bacterial test cultures of different concentrations are constructed based on fluorescence signals of AO-stained bacterial test cultures evaluated with the luminometer according to a protocol previously reported [18] for every parameter to be optimized.

### 2.5. Urine Samples Preparation and Detection of Pathogenic Bacteria

**Scheme 2:**
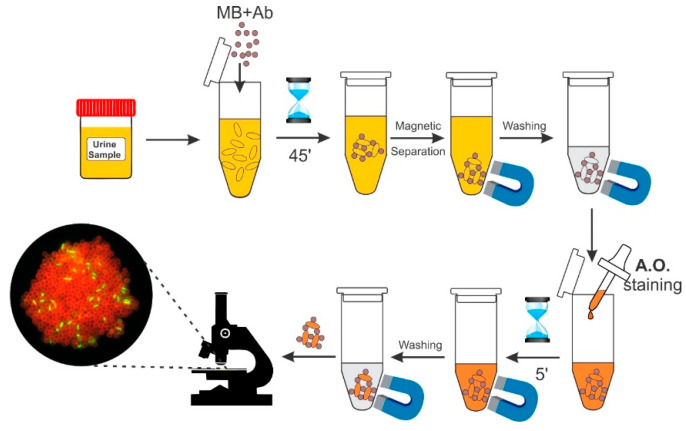
Steps of the protocol, from receiving urine samples to identifying pathogenic cells.

Unprocessed, anonymized urine samples (Synlab, Bucharest, Romania) are analyzed on the day of their reception. A total of 1 mL of the urine sample diluted 1:1 with PBS, pH 7.4, is mixed with 5 µL of MB stock, with MBs functionalized with different biorecognition compounds (antibodies or lectin). To facilitate target analyte binding on specifically functionalized MBs with an active biomolecule, the urine samples are incubated for 45 min under continuous mixing (rotary mixer) in 1.5 mL Eppendorf tubes at room temperature. After this step, the formed clusters/aggregates are separated from the sample by applying a magnet to the outside of the tube wall and washed three times in PBS and with PBS + 0.2% Tween 20 to reduce non-specific clustering. Subsequently, the microscopic analysis is performed by adding AO for staining (Scheme 2).

### 2.6. Fluorescence Microscopy

AO-stained MB clusters were deposited onto a glass microscope slide and covered with a microslide. Fluorescence and bright field images were recorded with an Eclipse 600, Nikon microscope (Nikon, Tokyo, Japan), 40× objective, a B2A fluorescence filter optimal for AO investigation, and a digital camera, Nikon D40X (Nikon, Tokyo, Japan).

## 3. Results

### 3.1. Optimization of Immunomagnetic Particles Synthesis Based on the Quantitative, Rapid Evaluation of the Capture Efficiency

The capture efficiency (CE) in IMS procedures is dependent on the quality of the immunomagnetic beads synthesis as well as on the dimension and number (i.e., MB/target cells ratio) of carrier beads used in the assay. The quality of the immunomagnetic beads is tailored by the type, concentration, and recognition potential of the affinity compound used in the functionalization of the immunomagnetic particles. A rapid fluorescence assay involving AO staining and the assessment of F_sup_ (the fluorescence of the cells that were not immunomagnetically separated) versus F_cells_ (the fluorescence of the cells undergoing immunomagnetic separation) was deployed to quantitatively evaluate these distinct parameters of immunomagnetic beads synthesis towards method optimization. To this end, specific calibration curves based on 10^8^, 10^7^, 10^6^, 10^5^, and 10^4^ CFU/mL bacterial test cultures are constructed based on fluorescence signals of AO-stained bacterial test cultures evaluated [18] with a luminometer (Table 1).

A total of 500 µL of each individual cell concentration is mixed with the optimized immunomagnetic particles (MBs functionalized with affinity compounds of various concentrations with or without prior orientation), incubated for different intervals (10, 15, 30, 45, and 60 min), and captured with a magnet on the vial’s side walls. The successful immunomagnetic separation (i.e., capture of the AO-stained cells with the formation of specific MBs clusters) corresponds to a decrease in the fluorescence signal of the supernatant and can be used to assess CE. C_sup_ is associated with F_sup_, the fluorescence of the supernatant that contains cells that were not immunomagnetically separated, and F_cells_ is the fluorescence of the original cell suspension spiked with specific bacteria at a controlled concentration (Table 1). The results represent the averaged data of three individual experiments, each with three fluorescence recording/measurement points. The background signal is around 50 FSU; the same time points were used for all fluorescence recordings to avoid AO fluorescence quenching effects. Accordingly, the specific calibration curves enable the precise quantitation of CE.

**Table 1:** Characteristic fluorescence signals of AO-stained suspensions of magnetically tagged bacterial cells of various concentrations (i.e., F_cells_).

Concentration (CFU/mL)	Fluorescence F_cells_ (FSU)
10^8^	632,053 ± 90
10^7^	71,861 ± 50
10^6^	8511 ± 30
10^5^	1105 ± 10

**Figure 1:**
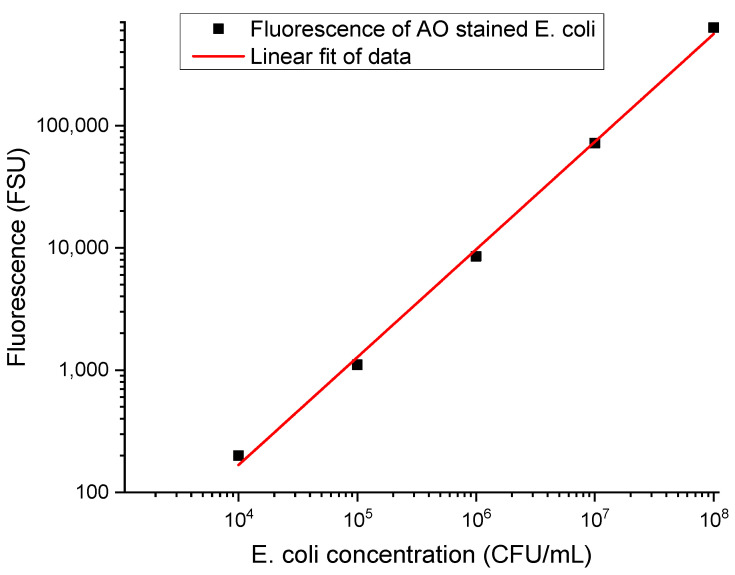
Calibration curve for suspensions of magnetically tagged cells AO-stained with the fluorescence signal as a function of the bacterial load.

The data in Table 1, corresponding to *E. coli* as a model strain for gram-negative bacteria, are used to derive useful calibration curves (Figure 1) for the characterization of the capture efficiency of the IMS protocols, but similar results can be acquired for gram-positive bacteria and fungi models (data not shown).

To quantitatively determine the capture efficiency of specifically functionalized MBs, the 10^6^ cells/mL concentration was selected as the reference. Growth media spiked with 10^6^ *E. coli* cells/mL are stained with AO, and their fluorescence is measured prior to (F_cells_) and after IMS (F_sup_) with specifically functionalized MBs, i.e., after the MB clusters were removed; only the supernatant (containing free cells) is assessed.

For the immunomagnetic material synthesis, the 2.8 μm dimension MBs were selected based on a previous study [18], and the 5 μL volume of immunomagnetic particles was selected in preliminary tests (data not shown) that were performed in parallel for MBs with carboxyl and tosyl (p-toluene sulphonyl) groups. As a rule of thumb, the smallest volume, giving, for both types of surfaces, a CE above 40%, was further considered in the optimization stages. To avoid interferences due to steric hindrances, all optimization steps involved the oriented binding of the affinity compound via protein G as an intermediate linker. Protein G’s major binding site in IgG is located in the Fc part of the antibody [26] and can be conveniently attached on materials’ surfaces with various activations.

Table 2 summarizes the optimization steps concerning the amount of immunoaffinity compound used for functionalization, the incubation time, and the characterization of the cross-reactivity, bacterial concentration, and live/dead status towards real sample tests.

Cross-reactivity was tested by incubating MBs coated with antibodies specific to *E. coli* with the specific target *E. coli*, the nonspecific gram-negative target Salmonella typhimurium, and the nonspecific gram-positive target Listeria monocytogenes. The results show adequate binding efficiencies of the MB-antibody complexes (Table 2) for both types of surface functionalization (carboxyl—CMB and tosyl—TMB). The high capture efficiency of both live and dead cells for tosyl-activated immunomagnetic material indicates caution for their use, without more optimization of the blocking conditions, in clinical samples due to a potentially high nonspecific capture and increased sensitivity to the matrix/urine composition.

Based on the equation and the F_cells_ and F_sup_ values in Table 1 provided by the fluorescence method (luminometer) using AO staining and optimized MB, one can estimate the capture efficiency for the IMS protocol for relatively high bacterial loads (concentrations). To confirm these data and evaluate the utility of captured cells in subsequent growth protocols (e.g., their relevance for the foreseen fast AST protocols on immunomagnetically enriched samples), the real-time recording of *E. coli* cells growth was performed using absorbance assays.

### 3.2. Optical Density Measurements and Real-Time Recording of E. coli Cells Growth

**Figure 2:**
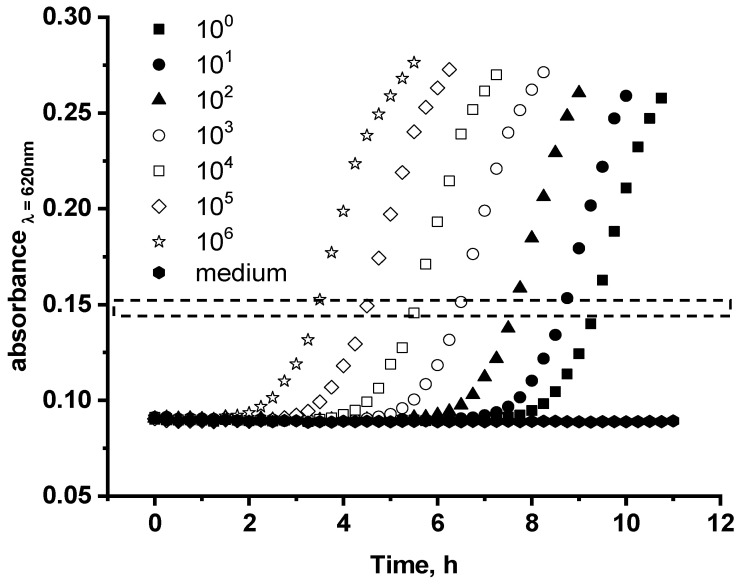
Growth dynamics of *E. coli* cells (starting concentrations 10^0^–10^6^ CFU/mL) in nutrient medium.

The relationship between the growth dynamics of bacterial cell suspensions as a function of the initial cell concentration and the capture efficiency of the newly synthesized materials for IMS, as part of process optimization, is evaluated through optical density measurements and the real-time recording of bacterial growth (*E. coli* chosen as the model). Suspensions of different *E. coli* concentrations were mixed (1:1) with a nutrient medium in a 96-well microplate and incubated for at least 12 h at 37 °C with shaking, and the corresponding absorbance values were recorded on a 15 min interval. Figure 2 reveals the characteristic growth dynamics of *E. coli* cultures, with starting concentrations in the range 1–10^6^ CFU/mL. For improved clarity, the growth curves highlight the lag and part of the exponential growth phases, and it is evident that the growth duration leading to a measurable signal that is robustly deviating from the baseline is inversely dependent on the concentration of the inoculum and can be used to derive the viable cell concentration with an appropriately constructed calibration curve.

**Figure 3:**
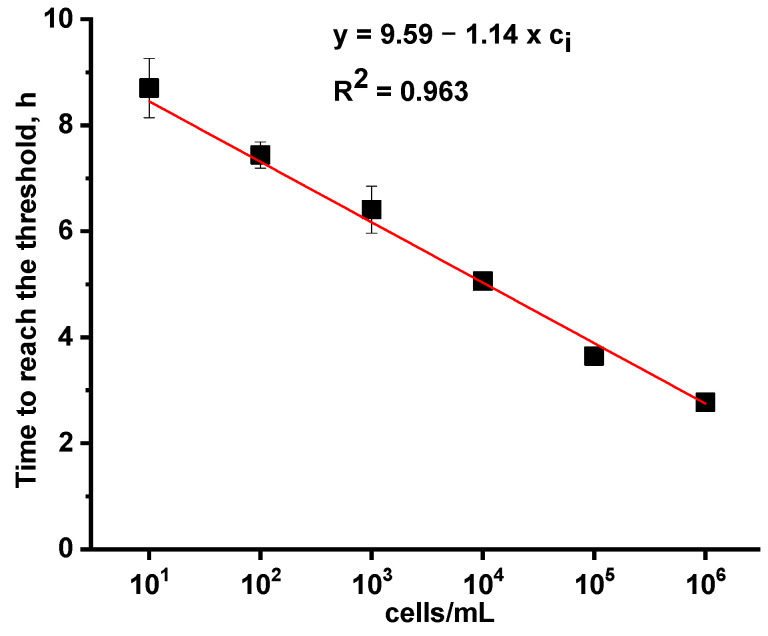
Calibration plot based on OD measurements (logarithmic representation) recorded in the concentration range of 10^1^–10^6^ cells/mL, indicated as c_i_. The exact durations are derived upon the automatic fit with a dose response function of every growth curve. Error bars indicate standard deviations for three independent measurements per initial cell concentration.

The time needed for each of the serially diluted *E. coli* suspensions to reach the same absorbance level/OD value (i.e., the same cell concentration) is derived based on growth curve fitting (dose response function) and can be used to obtain quantitative information from growth curves, even in the absence of plateau values that occur after long periods. We set this OD analytical threshold to 0.14 to ensure an appropriate signal/noise level for the optical density data of the actively growing cultures. The noise level in the OD determinations sets, in principle, the limit of detection, and the OD threshold can be lowered down to three times the noise level, with potentially faster detection/quantitation results. As shown in Figure 2, the time t_0_ (the time required to reach the set threshold—AL) varies in a manner that is inversely proportional with the initial cell concentration. For the highest concentration (10^6^ cells/mL), the cells reach AL after approximately 2 h, and for the lowest tested concentration in our set-up, it is achieved after more than 9 h. The calibration curve (Figure 3) derived from this time interval to reach the specific AL, evaluated in the concentration range of 10^1^–10^6^, was further used to determine the concentration of bacteria in samples with unknown bacterial loads and validate the capture efficiency. Monitoring *E. coli* cultures over time is especially useful for the low-concentration range, where direct evaluation is challenging, according to Table 1 data.

### 3.3. Optical Density Assay for the Evaluation of Captured Cells via the Real-Time Recording of E. coli Cells Growth

Similar to the fluorescence assay (Table 1) that indirectly provides the information on captured cells via the specific signal related to cells remaining in the solution upon IMS, the capture efficiency of the synthesized materials can be quantitatively assessed based on the growth curves of cells that are not immunomagnetically separated (e.g., the supernatant).

**Figure 4:**
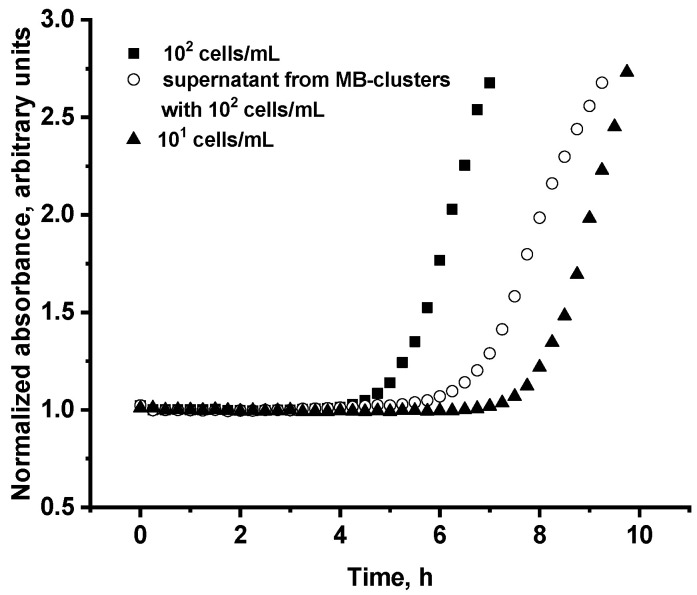
Cell growth profiles for *E. coli* cells assayed by recording the optical density of the samples (absorbance at λ = 620 nm). 10^2^ *E. coli* cells/mL, supernatant after the IMS of *E. coli* cells at a concentration of 10^2^ and diluted by ½ in the nutrient medium, 10^1^ *E. coli* cells/mL. Data referenced at the first value.

The IMS resulting supernatant was diluted by ½ in the growth medium, and 100 µL of this suspension was added in the microplate and evaluated via a time lapse absorbance assay. Moreover, after washing thoroughly three times with PBS, the collected MB clusters were added to 100 µL of the growth medium in the microplate to test whether the absorbance assay of the growth dynamics of cells collected in MB clusters can be also applied for the direct assessment of bacterial load (Figure 4).

We estimated growth rates in triplicate using the same conditions on different days.

The curve of the supernatant containing uncaptured cells in the IMS process shows a delayed growth compared with the growth curve of the unprocessed sample, with a time lag that is close to a 1:10 sample dilution. These data show that one can calculate the capture efficiency of the MB even at low target cell concentrations. Using the calibration curve (Figure 3), we estimated a CE of ~90% for this low concentration of cells.

**Figure 5:**
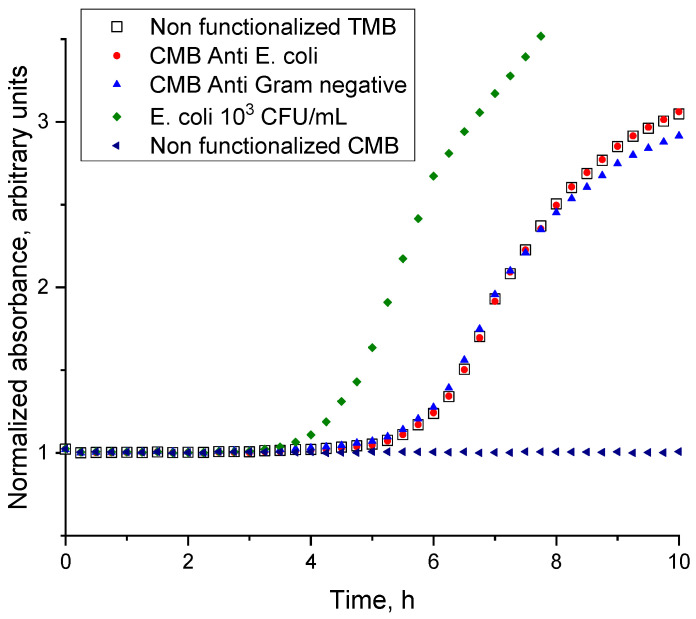
Growth curves of magnetically tagged *E. coli* upon capture from 10^3^ CFU/mL cell suspensions using CMB functionalized with Anti *E. coli* (red dots), Anti gram-negative antibodies (blue triangles), non-functionalized TMB (empty squares), and non-functionalized CMBs (arrow heads) compared with the growth curves of the untagged 10^3^ *E. coli* (green diamonds) cell suspension.

However, evaluating the growth dynamics of the cells undergoing IMS, i.e., the cells affinity-bound to the immunomagnetic materials using the same absorbance assay (Figure 5), highlights a modest cell load for every type of immunofunctionalization, which is indicative of the altered dynamics of the immunomagnetically captured cells (Anti-*E. coli* and Anti gram-negative synthetized materials). Accordingly, the absorbance assay of the growth dynamics of the cells collected in MB clusters (for cells immunomagnetically separated) must be approached with care when applied for the direct assessment of bacterial load.

Nevertheless, the growth of the cells captured by MB (Figure 5) shows that the bacteria are maintaining their viability after the capture and can be further analyzed and characterized by complementary methods.

The MBs were also tested for nonspecific adsorption, and the signal (Figure 5) showed the absence of bacterial growth for *E. coli* upon capture from 10^3^ CFU/mL cell suspensions using non-functionalized CMBs, which is indicative of a very low nonspecific binding as well as a high non-specific binding for TMBs. The growth curve of the magnetically tagged *E. coli* upon capture from 10^3^ CFU/mL cell suspensions using non-functionalized TMBs is similar to that of the magnetically tagged *E. coli* with CMB, with highly specific functionalization. The high nonspecific binding for tosylated beads (Table 2) is thus also confirmed in absorbance assays (Figure 5). Due to the observed non-specific binding of the MBs, experiments on real samples were conducted using only the carboxylated MB, with a mixture of AO staining and a direct, microscopy assay on IMS clusters.

### 3.4. Microscopy Assay for Urine Samples

Performing rapid, cost-, and labor-effective screening of clinical samples is highly desirable since, typically, of the total number of samples analyzed daily, more than half (~60%) are negative. Rapid screening in the urine sample can lead to a faster result, which can lead to a better medical decision for patients. It can also limit the unnecessary urine cultures.

While, from the clinical microbiology laboratory perspective, samples are characterized as negative when less than 10^3^ CFU/mL is present and positive for bacterial loads (of a single type of pathogen) above 10^5^ CFU/mL, when mixed populations are present at detectable levels, the samples are usually disregarded as non-valid and assumed to be contaminated.

The proposed procedure with the acridine orange staining of MB clusters formed in urine samples spotted on a microscope slide and examined under a fluorescent microscope yields characteristic images with cluster sizes, morphologies, and structure in a fast classification of bacterial load status.

**Figure 6:**
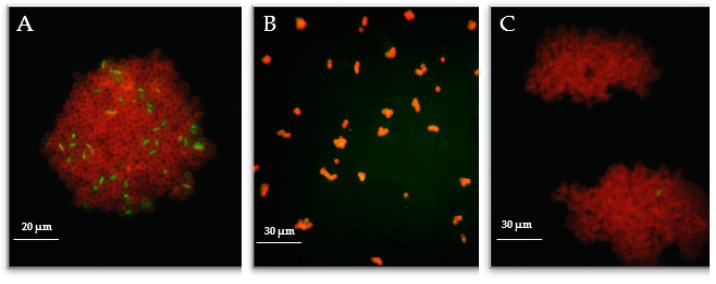
Characteristic images of MB clusters derived from urine samples: positive urine sample showing bacteria—*E. coli* captured with Anti *E. coli* MBs clusters (**A**), and negative urine samples with limited cluster formation (**B**) or with large nonspecific clusters with no bacteria present (**C**).

A sample was considered positive when bead aggregates were found and bacteria were evident in these clusters (Figure 6A). Similarly, for small sizes of clusters (Figure 6B) or no bacteria being present in otherwise large clusters (Figure 6C), the samples are considered negative.

According to clinical laboratory knowledge, most of the bacteria identified were gram-negative/*E. coli*; thus, the MBs functionalized with a generic antibody against gram-negative bacteria can be given preference in the direct testing of urine samples. However, cluster formation, as indicative of urine infection, was evaluated in parallel with MBs functionalized with an antibody against gram-positive bacteria and with MBL for wider recognition (images not shown). The high price of the last compound limits, to a certain extent, its applicability in routine analyses.

**Figure 7:**
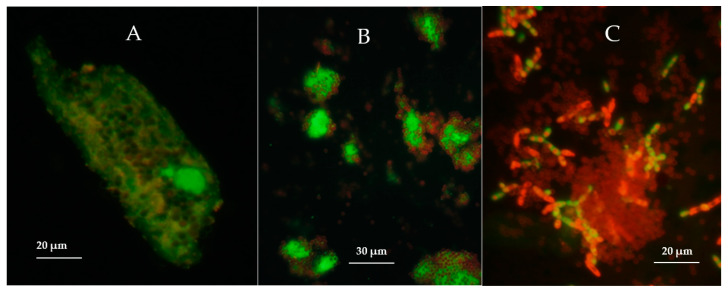
Nonspecific capture of non-target cells by MBL-MB. Epithelial cells present in urine samples—an epithelial cell covered with MB is shown (**A**), Mixed flora—clusters of bacteria—bright green—are surrounded by MB—dark red (**B**), Fungal presence—Candida spp. captured by the MBL-MBs (**C**).

Urine samples have notoriously variable compositions and matrix effects on IMS quality and, accordingly, cluster formation. The urine samples are 1:1 diluted in PBS buffer and undergo IMS without further purification steps. Characteristic images of nonspecific clusters formed due to the presence of epithelial cells (Figure 7A) or the highly heterogeneous clusters for mixed flora (Figure 7B,C) are worth highlighting (images obtained when using MB functionalized with MBL). These cases are nevertheless easy to discriminate—epithelial cells and fungi are bigger and can be distinguished from bacteria; moreover, typically, the mixed flora samples are deemed as non-compliant and are rejected by the test laboratory.

**Figure 8:**
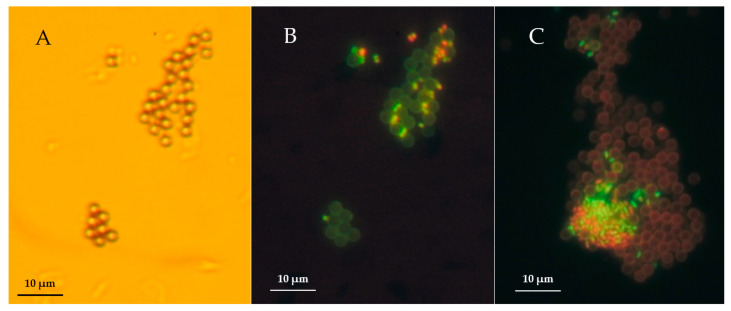
Comparison of transmission (**A**) and fluorescence microscopy (**B**) images of aggregates of MB, with Anti-*E. coli* O & K functionalization, mediated by captured *E coli* bacteria. Example of a “false positive” sample (**C**) according to clinical laboratory data, i.e., bacteria mediated clustering for pathogens at concentrations below the positivity threshold set by the clinical laboratory.

AO staining adds tremendously in terms of discriminatory power, as evident in Figure 8, when comparing the transmission and fluorescence images of MBs with Anti-*E. coli* O & K functionalization. Bacteria can be unequivocally identified against MB only in fluorescence images. Moreover, effective cluster formation, visible bacteria (Figure 8B,C), and, hence, positive samples according to microscopy standards are demonstrated even for concentrations lower than the clinical positivity threshold for bacterial load in urine; however, in the further analysis, these types of results (Figure 8C), if occurring, are named “false positive” in order to enable a comparison to the clinical laboratory results for the validation and evaluation of the method’s performance.

### 3.5. Method Performance

**Figure 9:**
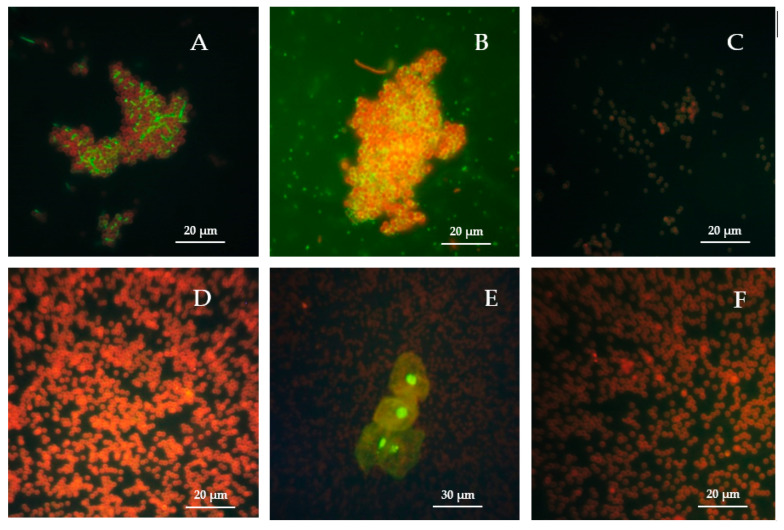
Examples of test kit results on urine samples: (**A**–**C**) test kit 1 assay of a clinical sample with a dual catheter infection: *Proteus* and *Enterococcus*. Anti gram-negative functionalized MB—green-stained bacteria are present in large clusters (**A**); Anti gram-positive functionalized MB—yellow-green-stained bacteria are present in large clusters (**B**); reference non-functionalized MB—no clusters and no bacteria present (**C**). (**D**–**F**) Test kit 2 sample—negative; Anti-*E. coli* MB—no clusters and no bacteria present (**D**); MBL MB—no clusters and no bacteria present, although epithelial cells are present (**E**); reference non-functionalized MB—no clusters and no bacteria present (**F**).

Testing real urine samples is performed with multiple referencing, as enabled by the optimized diverse MB functionalization: test set 1—Anti gram-positive, Anti gram-negative, or Anti *E Coli* antibodies, and test set 2—MBL and Anti *E. Coli* antibody. Examples of the images obtained in the positive and negative samples are presented in Figure 9, with nonfunctionalized MBs used as the urine sample quality reference and for assessing nonspecific clustering. Convenience, test relevance, and generic gram-specificity recommended that the test set 1 composition be further used to assess the method’s performance.

The performance of our method was evaluated by individually comparing the positive and negative samples according to the microscopy assays of AO-stained MB bacterial clusters with test set 1 with the qualitative results provided with reference methods in clinical laboratory tests and performing the related statistical analysis.

Statistics associated with the method’s specificity and sensitivity are defined according to the standard definitions:

$$Specificity\;\left(\%\right)=\frac{N\#\;of\;true\;negative\;results\times 100}{N\#\;of\;true\;negative\;results+N\#\;of\;false\;positive\;results}$$

and

$$Sensitivity\left(\%\right)=\frac{No\;of\;true\;positive\;results\times 100}{\left(No\;of\;true\;positive\;results+No\;of\;false\;negative\;results\right)}$$

A total of 58 urine samples were analyzed using our method. Of these, 29 were found to be true negative, 23 were found to be real positive, 5 were found to be false positive, and 1 was found to be false negative (Table 3). The positive samples were further processed by the clinical lab and were found to contain bacteria from the species *E. coli*, *Klebsiella pneumoniae*, *Pseudomonas* spp., *Proteus* spp. (gram-negative), *Enterococcus*, and *Streptococcus beta hemolytic* group b (gram-positive). Given the high specificity (85.29%) and sensitivity (95.83%), our method is demonstrated to be suitable for providing rapid results within the primary care setting for emergency UTIs.

## 4. Discussion

The culture-based method remains the gold standard for the detection of (pathogenic) bacteria and even for the evaluation of the capture efficiency of most IMS procedures, with turbidity, as determined by optical density or dye indicators (either colorimetry or fluorescence) and individual cell counts (as in plate count methods), as the preferred assays. Moreover, point of care (POC) microscopy, either to detect Pyuria (urine examined through a microscope for the presence of white blood cells; samples may be centrifuged before examination) or *Bacteriuria* (urine examined for the presence of bacteria; urine may be Gram stained), can potentially become the standard for urine analysis in time-sensitive situations, despite the fact that, currently, there is no evidence-based consensus regarding the use of urinary microscopy in general practice [27]. It is a more convenient approach compared to other methods (e.g., dipslide and rapid culture methods, colorimetric tests, impedance, bio- and chemical luminescence, immunologic tests (e.g., ELISA), enzyme tests, bacterial oxygen consumption) that currently meet a limited acceptance in clinical laboratories. It enables the clinician to determine the morphology of the organisms (rod or cocci) and describe their type of motility (non-motility, polar, or non-polar motility) in addition to quantifying the number of bacteria seen per field of vision. As an overall note, pre-enriched and/or spiked samples are typically used [28,29]. Moreover, while detection methods are described in the literature (Table 4) to provide the rapid detection of pathogens, due to the complexity of urine matrices, their standardization and direct application on clinical samples are very difficult.

The objective of this study was to develop and optimize an analytic protocol, based on specific cluster formation upon IMS, that does not require any sample pre-treatment for the rapid detection of the presence of uropathogens in urine samples. This was achieved via materials optimized for immunomagnetic separation and a microscopy assay of the formed clusters due to the presence of target pathogens. The protocol enables subsequent access to the bacterial culture for further phenotypic analysis (i.e., preserving cell viability).

In a previous study [18], we showed that the selective capture of the target analyte and the formation of cell clusters can be revealed by measuring the oscillations of magnetically labelled analytes when applying a periodic magnetic field. A dependence of the incubation time as well as on the limit of detection was demonstrated in conjunction with the dimension of the MBs, with the 2.7 μm size providing the best performances.

We built on this knowledge and we optimized test MBs sets for immunomagnetic selection (IMS) and specific cluster formation in clinical urine samples.

Gram-negative bacterial infections appear to dominate the UTI spectrum, with many of the bacterial infections reported to be caused by *E. coli*. However, the spectrum of causative agents in UTIs is wide, with both gram-positive bacteria and fungi potentially present. The bio-affine functionalization of magnetic beads is essential for the successful immunocapture. Therefore, the study approached MB functionalization towards IMS in urine with a larger range of affinity compounds. In addition to both highly specific antibodies (to easily identify the type of pathogens present in the urine samples) as well as compounds with wide affinity (e.g., anti gram-type) antibodies, we tested, for immunomagnetic material synthesis, the specific recognition based on mannan-binding lectin (MBL). MBL recognizes carbohydrate patterns found on the surface of a large number of pathogenic micro-organisms, including bacteria, viruses, protozoa, and fungi. MBL is a protein belonging to the collectin family that is produced by the liver and can initiate the complement cascade by binding to pathogen surfaces. Diverse *Candida species*, *Aspergillus fumigatus*, *Staphylococcus aureus*, and beta-hemolytic group A streptococci exhibit the strong binding of MBL, whereas *Escherichia coli*, *Klebsiella* species, and *Haemophilus influenzae* type b are characterized by heterogeneous binding patterns. In contrast, beta-hemolytic group B streptococci, *Streptococcus pneumoniae*, and *Staphylococcus epidermidis* show low levels of binding [30,31,32].

To increase the discrimination potential of our method, we used Acridine Orange (AO) staining. AO is a fluorochrome stain used for live cells and bacteria. The staining mechanism is particular for DNA (intercalation interactions) and for RNA (mostly via electrostatic interactions) [33]. AO is potentially superior to the Gram stain in the direct microscopic examination of clinical specimens because it gives striking differential staining between bacteria and background cells and debris [34] and was proven to be [21,33] fast, accurate, inexpensive, and effective for various synthetic and clinical (urine) sample analyses; hence, it is useful in UTIs.

Immunomagnetic selection, in combination with dynamic assessment, was progressively optimized, starting with synthetic samples. The results presented in Figure 2, Figure 3, Figure 4 and Figure 5 show that the quantitative evaluation of capture efficiency is enabled by AO fluorescence and absorbance measurements of growth dynamics to support the optimization of the IMS protocol. The procedure can be also applied to immunomagnetically separated fractions (e.g., the supernatant, as well as to cells collected in MB clusters) and even to cells washed from the clusters subsequent to repeated washing steps, if needed, when optimizing various protocols.

While the capture efficiency is a suitable parameter to support protocol optimization, our study revealed some significant details: (a) the capture efficiency is dependent on the target cell concentration domain, (b) a high capture efficiency might be detrimental for highly complex matrices (such as urine), (c) cell growth can be evaluated at the analytic threshold (100 cells) via classical OD assays in hours, (d) the growth kinetics for cells trapped on the MB and of cells free in the solution are not fully similar.

The microscopic analysis of immunomagnetic clusters, in conjunction with AO staining and generic bioaffine functionalization (antibodies against the gram-specific cell wall components Lipoteichoic acid (LTA) and gram-negative Endotoxin, and mannan-binding lectin recognizing carbohydrate patterns found on the surface of a large number of pathogenic microorganisms), was demonstrated to be an effective solution for the direct, rapid detection of bacterial load in urine samples. However, MB cluster formation is the sole process with analytic relevance, while AO staining is a mere indicator of the cluster formation specificity. Moreover, our data demonstrate that the use of AO staining alone only allows for the quantitation of capture efficiency in a concentration domain above the relevant clinical threshold (10^4^–10^5^ cell/mL), challenging the plethora of studies in the literature building AO-mediated fluorescence assays.

A total of 58 urine samples were analyzed using our method. The results obtained on the affinity-mediated clusters in unprocessed urine samples were compared with those of the conventional culture-dependent method, and the developed method was proven to be useful in identifying and assessing the presence of pathogens in urine samples in approximately 60 min, with a high specificity (85.29%) and sensitivity (95.83%). As such, it provides significant progress beyond the reported assays (Table 4).

The limitations of the technique could reside in MB clustering in the absence of bacteria. However, the use of AO staining and the tailored test kits enables multiple referencing, eliminating, to a large extent, this issue.

Since the IMS method and procedure preserve the viability of the cells that are immunomagnetically enriched, the method could be conveniently extended towards the development of rapid AST platforms, enabling the evaluation of the bacterial cell division under antimicrobial conditions and, hence, drug resistance.

## 5. Conclusions

The problem of the direct, rapid detection of pathogens from clinical samples related to urinary tract infections poses significant analytical challenges. While indirect methods (e.g., urine microscopy or dip sticks) are often used in practice to assess the presence/absence of bacteria or inflammation, the gold standard for diagnosing UTIs in the presence of clinical symptoms is the identification and quantification (determination of bacteriuria) of the pathogen via the urine culture, a procedure both time- and resource-consuming. We address these issues via materials optimized for immunomagnetic separation and a microscopy assay of immunomagnetic clusters encompassing target pathogenic cells and magnetic beads functionalized with bio-affine coatings. These bio-affine coatings were tailored for the specific UTI pathogens to include a wide range of compounds, from antibodies against *E. coli* or against gram-specific cell wall components to mannan-binding lectin recognizing carbohydrate patterns found on the surface of a large number of pathogenic microorganisms, including bacteria, viruses, protozoa, and fungi. The affinity-coated magnetic beads play a double role—for capturing the target pathogenic cells from the bio-sample and for mediating the formation of aggregates of specifically bound pathogens and magnetic particles, the latter allowing for a straightforward assessment of the bacterial load via optical analysis.

Analyzing the structure of the aggregates of magnetically tagged pathogens from minimally processed urine samples, the assay provides, in less than 60 min, qualitative (positive/negative) information on the bacterial load. The 85% specificity and 96% sensitivity of the assay are achieved by optimizing the immunomagnetic material, the selection of generic anti-gram-negative antibodies and mannan binding lectin, as well as the use of nonspecific, inexpensive fluorescent dye, enabling clinical microscopy with a new analytic dimension, prone to automation. The limitations of the technique could reside in MB non-specific self-clustering in the absence of bacteria, especially in complex matrices; however, as demonstrated in the study, upon careful design of the materials for immunoseparation and the use of the fluorescence assay on clusters, it is possible to perform accurate analyses in urine. This study proposes an effective sensing protocol to analyze real urine samples that can be conveniently used in clinical microbiology laboratories for the prescreening of samples. Moreover, the study emphasizes a suite of practical validation assays when developing magnetic materials for immunocapture applications. The detection procedure preserves the viability of the captured bacteria and, as a future avenue, can be interfaced with downstream analyses and antimicrobial susceptibility testing.

## Figures and Tables

**Table 2:** Summary of optimization steps.

**Amount of immunoaffinity compound (μg)** **(carboxyl MB)**	0.5		1		2		5	
Estimated CE%	50		45		41		39	
**Incubation time (min)** **(carboxyl MB)**	10		15		30		45	
Estimated CE%	29		35		42		60	
**Type of MB surface** **Functionalization with 0.5 μg**	Carboxyl (CMB)				Tosyl (TMB)			
Estimated CE%	>65%				>75%			
**Live/dead status and cell concentration**	Carboxyl (CMB)				Tosyl (TMB)			
**Cell concentration**	10^5^		10^6^		10^5^		10^6^	
**Cell status**	dead	alive	dead	alive	dead	alive	dead	alive
Estimated CE%	0	60	21	61	27	75	56	78
**Cross-reactivity** **(CMB + Anti *E. coli*)**	*E. coli*				Nonspecific *Listeria mon*.		Nonspecific *Salmonella typ.*	
Estimated CE%	60				3		12	

**Table 3:** Results obtained from the analysis of urine samples. Comparison with the method performed in a clinical laboratory.

	Positive	Negative	False Positive	False Negative	True Positive	True Negative
Our method	28	30	5	1	-	-
Clinical lab	24	34	-	-	-	-
	**Statistic data**					
Specificity	85%		5	-	-	29
Sensitivity	96%		-	1	23	-

**Table 4:** Examples of different methods (marked with bold font) for *E. coli* and other bacteria detection.

Technology/Method	UTI Pathogens	Complexity	Time to Result (min)	Direct Detection from Clinical Samples	Ref.
**Microscopic Screening** Specific clusters formation upon immunomagnetic capture; Confirmatory Acridine orange staining	Yes (gram-positive and gram-negative bacteria, some fungi)	- cheap, quick and requires only limited technical skill - eliminates the need for cultures - minimal sample preparation - captured bacteria are viable and can be recovered and cultivated for further analyses	45	Yes (uncentrifuged urine samples)	Our work
**Microscopic Screening** Non-specific Acridine orange staining	Yes (non-specific)	- rapid and cost-effective - non-specific	N/A	Yes (smears)	[34]
**Immunological-based** **Assays** (RapidBac test—a lateral flow immunoassay test strip)	Mostly gram-negative bacteria	- similar to a home pregnancy test - rapid, does not require sample processing The test utilizes a cocktail of monoclonal antibodies targeting a panel of bacterial surface proteins	20	Yes	[5]
**Molecular method** Real-time PCR (molecular beacon–Au nanoparticle)	*E. coli*	- easy preparation and low cost - 10^3^ times more sensitive than traditional beacon probes	<60	Clinical urine samples with 3 × 10^7^ CFU/mL	[23]
**Molecular method** Centrifugal chip based on DNA extraction, multiplex recombinase polymerase amplification, and fluorescent detection	*E. coli, S. aureus, S. typhimurium, P. mirabilis*, and *P. aeruginosa*	- laborious technique	<40 min	No—spiked-urine samples	[24]
**Microfluidic platform** Bead-based biosensor via fluorescence imaging	*E. coli*	- semi-automated system and relatively easy to operate	1 h	No—spiked urine samples at a concentration of 5 × 10^4^ CFU/mL	[22]
SPR biosensor (surface imprinting with Au NPs)	*E. coli*	- rapid and real-time detection	-	No—artificial urine-spiked *E. coli*	[35]
**Fluorescence microscopy** system based on magnetic glycol-particles	*E. coli*	- rapid detection without time-consuming cell culturing - capture efficiency was 88%	45 min	No—*E. coli* in buffer	[9]
**IMS and fluorescence techniques** (QDs nanoparticles)	*E. coli*	- rapid detection compared with PCR methods - laborious work; many steps are required	<120 min	No—*E. coli*-spiked urine samples	[10]
**Fluorescent nanoparticle-based indirect immunofluorescence** microscopy fluorescence	*M. tuberculosis*	- higher luminescence and higher photostability - can be a universal method for detecting a wide variety of bacteria	240	No—mixed bacteria and spiked sputum samples	[36]

LFI—lateral flow immunoassay; QD—Quantum Dots; Ab-MNP—antibody-conjugated magnetic nanoparticles; CE SSCP—capillary electrophoresis single-strand conformational polymorphism; MLPA—multiplex ligation-dependent probe amplification; IMS—immunomagnetic separation.

## Data Availability

The data generated during the present study are available from the corresponding authors on reasonable request.
